# Mitigating disuse‐induced skeletal muscle atrophy in ageing: Resistance exercise as a critical countermeasure

**DOI:** 10.1113/EP091937

**Published:** 2024-08-06

**Authors:** James McKendry, Giulia Coletta, Everson A. Nunes, Changhyun Lim, Stuart M. Phillips

**Affiliations:** ^1^ Exercise Metabolism Research Group, Department of Kinesiology McMaster University Hamilton Ontario Canada

**Keywords:** anabolism, catabolism, muscle unloading, physical activity, sarcopenia

## Abstract

The gradual deterioration of physiological systems with ageing makes it difficult to maintain skeletal muscle mass (sarcopenia), at least partly due to the presence of ‘anabolic resistance’, resulting in muscle loss. Sarcopenia can be transiently but markedly accelerated through periods of muscle disuse‐induced (i.e., unloading) atrophy due to reduced physical activity, sickness, immobilisation or hospitalisation. Periods of disuse are detrimental to older adults' overall quality of life and substantially increase their risk of falls, physical and social dependence, and early mortality. Disuse events induce skeletal muscle atrophy through various mechanisms, including anabolic resistance, inflammation, disturbed proteostasis and mitochondrial dysfunction, all of which tip the scales in favour of a negative net protein balance and subsequent muscle loss. Concerningly, recovery from disuse atrophy is more difficult for older adults than their younger counterparts. Resistance training (RT) is a potent anabolic stimulus that can robustly stimulate muscle protein synthesis and mitigate muscle losses in older adults when implemented before, during and following unloading. RT may take the form of traditional weightlifting‐focused RT, bodyweight training and lower‐ and higher‐load RT. When combined with sufficient dietary protein, RT can accelerate older adults' recovery from a disuse event, mitigate frailty and improve mobility; however, few older adults regularly participate in RT. A feasible and practical approach to improving the accessibility and acceptability of RT is through the use of resistance bands. Moving forward, RT must be prescribed to older adults to mitigate the negative consequences of disuse atrophy.

## Abstract (highlights)

- **What is the topic of this review?**
  Use of resistance exercise training to prevent or mitigate the disuse‐induced muscle atrophy in an ageing population.
- **What advances does it highlight?**
  The review highlights several potential mechanisms of disuse‐induced muscle atrophy and strategies before, during and following disuse to prevent, mitigate and recover lost muscle.

## 1 INTRODUCTION

Ageing is a complex process marked by myriad physiological changes. One prominent aspect of ageing is the decline in muscle mass (Mitchell et al., 2012; Nunes et al., 2022)—sarcopenia (Cruz‐Jentoft et al., 2019)—and functional mobility, which presents a monumental challenge to social and healthcare systems. Sarcopenia increases the risk for a plethora of adverse health outcomes—falls (Cawthon et al., 2019), frailty (Greco et al., 2019), hospitalisation (Beaudart et al., 2014), non‐communicable diseases (Chaput et al., 2023) and early mortality (Xu et al., 2022)—and cost the UK and Canada ∼$2.5–3 billion each in 2021 (Chaput et al., 2023; Pinedo‐Villanueva et al., 2019). Concerningly, episodic periods of muscle disuse can punctuate and amplify sarcopenic decline (Nunes et al., 2022; Wall et al., 2016), accelerating the trajectory towards disability and physical dependency (English & Paddon‐Jones, 2010). In a society where physical activity levels are declining with age, ∼85% of older adults do not meet current physical activity guidelines (Bennie et al., 2019; Colley et al., 2011), and sedentariness is increasing. For example, ∼67% of older adults spend >8.5 h/day being sedentary (Copeland et al., 2017; Harvey et al., 2013), and consequent muscle mass and strength losses are a serious cause for concern. However, the trajectory of muscle loss is modifiable, and strategies to mitigate the harmful effects of disuse are the focus of intense scientific inquiry. Regular engagement in physical activity and exercise training—particularly resistance training (RT)—and various nutritional interventions may offset musculoskeletal deterioration and potentially reduce the risk of several non‐communicable diseases (McLeod et al., 2019; Pedersen & Saltin, 2015). Thus, the clinical and financial value of addressing low skeletal muscle mass and strength losses with scalable interventions is obvious and should be a primary focal point to promote healthy and independent ageing.

## 2 MUSCLE PROTEIN TURNOVER, ANABOLIC RESISTANCE AND DISUSE

Skeletal muscle ‘senses’ a complex interplay of mechanical (i.e., via loading/unloading or aerobic exercise) and molecular (i.e., substrate availability as amino acids, glucose or lipids) perturbations and integrates a series of protein signalling events to modify the rates of muscle protein synthesis (MPS) and muscle protein breakdown (MPB) (McKendry et al., 2021). The algebraic difference between MPS and MPB determines the net protein balance within muscle (Phillips, 2004) and, thus, whether muscle proteins are accrued or degraded. MPS and MPB oscillate throughout the day in response to feeding and fasting. In young, healthy individuals, protein ingestion leading to hyperaminoacidaemia stimulates MPS (Moore et al., 2009; Trommelen et al., 2023). RT also serves as a robust stimulus to increase MPS, which works additively with protein feeding to stimulate MPS beyond either stimulus alone (Biolo et al., 1995, 1997). It can be problematic to infer changes in muscle mass from acute MPS studies (Witard et al., 2021); however, Damas and colleagues have elegantly shown a time course‐dependent relationship between the acute response of MPS to RT and chronic changes in muscle mass during RT (Damas et al., 2016), which is modified by training status. However, the muscles of older individuals gradually become less sensitive to anabolic stimuli (e.g., exercise and protein nutrition) (Brook et al., 2016; Kumar et al., 2009; Moore et al., 2015), which is an age‐related phenomenon known as anabolic resistance and can make maintaining skeletal muscle mass with ageing especially challenging. Muscle disuse, which is highly prevalent in older individuals, induces anabolic resistance (Wall et al., 2016).

Short‐term intermittent muscle disuse—sedentariness (i.e., reduced number of daily steps), unilateral limb immobilisation (ULLI) or bed rest—occur throughout life and lead to rapid and considerable muscle atrophy (Hardy et al., 2022), which is, especially with advancing age, difficult to recover. Current evidence suggests that reduced postabsorptive (Gibson et al., 1987; Glover et al., 2008; Symons et al., 2009) and postprandial (Glover et al., 2008; Wall, Snijders et al., 2016, 2013) MPS underpin disuse‐induced atrophy, as opposed to increased MPB (Brook et al., 2022; Stokes et al., 2020; Symons et al., 2009; Wall, Dirks et al., 2013). ULLI reduces integrated‐MPS (∼9.5–36%) (Kilroe et al., 2020; Stokes et al., 2020) and induces losses of knee extensor muscle size and strength by ∼5–14.5% and ∼23%, respectively (Preobrazenski et al., 2023). Whilst the rate of atrophy may differ slightly between muscles and the instigating disuse event, a recent pooled analysis revealed that the quadriceps muscle size typically declines at ∼0.46%/day in response to unloading—with more compromised populations (i.e., critical care) experiencing losses ∼2.5 times faster (Hardy et al., 2022). Disuse‐induced muscle loss slows to 0.33%/day over 28 days (Hardy et al., 2022), aligning with prolonged bed rest interventions (∼25% in 60 days) (Trappe et al., 2007). A recent analysis, albeit with a small sample size, indicates the amount of muscle an individual has before the disuse event does not influence the rate of muscle loss; however, sex‐based differences may warrant further investigation (Coffey et al., 2023). There is also emerging evidence that anatomical location or fibre type may result in differential rates of muscle loss (Bass et al., 2021).

Regardless of the rate of muscle loss, older individuals beginning a disuse event with more muscle would have a greater muscle mass ‘buffer’ to withstand periods of unloading that may allow them to avoid more dire consequences of disuse (English & Paddon‐Jones, 2010). Upon remobilisation, young, healthy individuals recover lost muscle mass and strength with relative ease—returning to habitual daily activities is sufficient to recover muscle mass and function (Suetta et al., 2009b). However, as building new muscle is slower (∼5 times) than the rate at which it is lost (Stokes et al., 2020), the presence of anabolic resistance in older individuals means they do not possess the same resilience and recovery potential as their younger counterparts (Suetta et al., 2009b). Subsequently, older individuals may fail to regain lost muscle and experience prolonged loss of muscle strength and compromised physical function, which increases mortality risk and worsens quality of life (Atherton et al., 2016; Larsson et al., 2019a). Thus, the need for targeted interventions to augment muscle mass to counteract inevitable periods of muscle disuse is clear.

## 3 MECHANISMS OF MUSCLE LOSS WITH AGEING AND DISUSE

**FIGURE 1:**
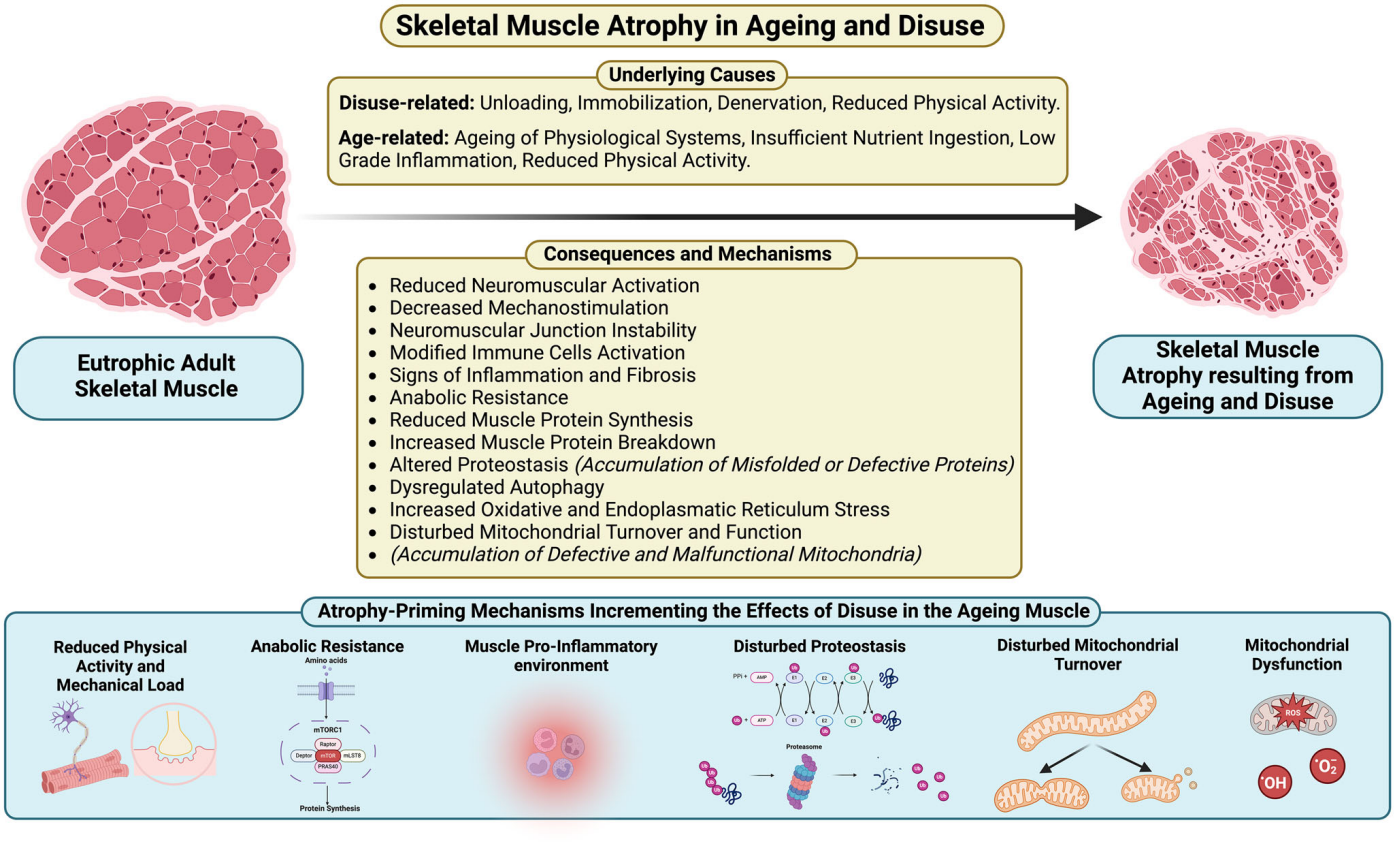
Schematic depiction of the consequences and mechanisms of ageing‐ and disuse‐induced skeletal muscle atrophy (Chen et al., 2018; Deane et al., 2021; Fix et al., 2021; Larsson et al., 2019a; Paez et al., 2023; Spendiff et al., 2016). Created with BioRender.com.

The mechanisms underpinning muscle loss with ageing and disuse are complicated, and the timeline of sarcopenia makes it challenging to determine the primary mechanism (outlined in Figure 1). MPS, MPB and occasional inactivity could all play progressive roles in disuse‐induced muscle loss (Nunes et al., 2022). Decreases in daily physical activity (Takagi et al., 2015) and alterations in the systemic levels of hormones and pro‐inflammatory cytokines that occur with ageing contribute, to a variable degree, to age‐related muscle loss (Santoro et al., 2021) by decreasing anabolic responses. In older adults, MPS stimulation in response to anabolic stimuli is diminished compared to younger adults despite similar basal protein synthesis rates (Phillips et al., 2009; Wall et al., 2016). Basal mechanistic target of rapamycin complex 1 (mTORC1) activity, the primary signalling axis which increases MPS in response to amino acids (McGlory, Nunes et al., 2018), is greater in older adults than younger adults (Markofski et al., 2015). Sustained activation of the mTORC1 signalling axis may impair the subsequent pathway activation, leading to a global decrease in the system's sensitivity to anabolic inputs (Atherton et al., 2016; Wall et al., 2016). In small animal models, previous work from David Glass's group has shown that partial inhibition of mTORC1 ameliorated the decline in skeletal muscle mass associated with ageing and may hold promise as a therapeutic target (Joseph et al., 2019). On top of such anabolic resistance to amino acids, older adults often do not consume sufficient protein and essential amino acids to fulfil muscle requirements (Coelho‐Junior et al., 2022), and the insulin‐mediated suppression of muscle protein breakdown appears attenuated in older adults (Atherton et al., 2016).

Chronic low‐grade inflammation is another recognised factor in the age‐related decline of muscle health (Santoro et al., 2021). Immune cell activation status (e.g., pro‐inflammatory macrophages) in disuse‐induced atrophy is important in regulating muscle homeostasis and remodelling in ageing (Fix et al., 2021). Chronic low‐grade inflammation in older individuals may disguise the necessary inflammatory transcriptional signatures induced by stressors like RT (Deane et al., 2021). Fibrosis, a hallmark of chronic inflammatory processes, is a common finding in the muscles in preclinical models of ageing and sarcopenia (Shang et al., 2020). Additionally, macrophage‐mediated myogenesis and macrophage activation states are altered in the muscles of preclinical models of ageing (Deane et al., 2021; Fix et al., 2021). Thus, chronic inflammation in older individuals—so‐called ‘inflammageing’—may partly underpin the hindrance of muscle regenerative capacity and growth in response to anabolic stimuli (Ferrara et al., 2022).

Some have suggested that disuse induces increased oxidative stress and that this may contribute to disuse atrophy and that this is a target for antioxidant (AOx) supplementation to alleviate atrophy (Powers, 2014). Whilst rodent models have shown some success in mitigating atrophy using AOx cocktails, human data are mixed, and AOx cocktails have shown limited (Damiot et al., 2019) or no protective effects against disuse atrophy (Arc‐Chagnaud et al., 2020; Noone et al., 2023).

Nonetheless, the loss of proteostasis is a distinctive characteristic of ageing skeletal muscle, differentiating it from disuse (Paez et al., 2023). Ageing is linked with the buildup of damaged or dysfunctional proteins and organelles (e.g., mitochondria) (Paez et al., 2023). This decline in proteostasis appears to be fibre‐type specific, with type 1 fibres showing an increase in components of protein synthesis and ubiquitin–proteasome system machinery, as well as chaperone complexes, whilst type 2A fibres show a decrease in these proteins (Ayyadevara et al., 2016). These findings align with the preferential atrophy of type 2 fibres with ageing (Murgia et al., 2017). As autophagy and mitophagy are also decreased in muscle during ageing (Leduc‐Gaudet et al., 2021), muscle function may also be compromised due to the presence of non‐autophagocytosed mitochondria and other damaged cellular components (Chen et al., 2018). Dysfunctional mitochondria tend to accumulate within lysosomal lipofuscin bodies in the muscles of older mice (Chen et al., 2018). Likewise, older animals show a heightened emission of mitochondrial reactive oxygen species, contributing to organelle damage (Gouspillou et al., 2014; Sonjak et al., 2019). Transcriptional signatures associated with mitochondrial turnover also decrease in older adults, accumulating dysfunctional mitochondria (Drummond et al., 2014; Leduc‐Gaudet et al., 2021) and exacerbating deficits in mitochondrial energetics (Mau et al., 2024, 2023).

Physical inactivity contributes to impairments in mitochondrial respiratory function with increasing age (Spendiff et al., 2016); however, in advanced age (>75 years), the accumulation of denervated fibres—due to failed denervation–reinnervation cycles—results in further mitochondrial impairment (Spendiff et al., 2016) that may induce myofibre atrophy and loss through apoptotic processes (Alway et al., 2017). Preclinical data show that, during ageing, most neuromuscular junctions (NMJ) remain stable, whilst a small fraction undergo rapid and transformative changes, losing original acetylcholine receptor labels and acquiring fragmented patterns. This stochastic transformation occurs independently of the initial NMJ appearance, suggesting a dynamic process during ageing that likely favours net protein degradation and muscle fibre loss (Larsson et al., 2019b). Human investigations, however, have demonstrated that short‐term muscle disuse induces notable changes in neuromuscular properties—reduced motor unit potential and firing rate (Inns et al., 2022), increased NMJ instability and impaired sarcoplasmic reticulum calcium handling (Monti et al., 2021). Overall, the evidence suggests a complex process involving continuous remodelling, compensatory reinnervation and focal denervation, highlighting the intricate dynamics of NMJ changes during ageing (Larsson et al., 2019b).

Finally, preclinical data comparing muscle atrophy in adult males and females showed distinct responses to simple disuse (Rosa‐Caldwell, Lim, Haynie, Brown, Deaver et al., 2021). Female mice seem to preserve mitochondrial function at the expense of muscle mass during the early days of disuse‐induced muscle atrophy (Rosa‐Caldwell, Lim, Haynie, Brown, Lee et al., 2021). The increased activation of Deptor and Redd1 in female mice, especially in fast fibre‐rich muscle, corroborates the increased vulnerability of type 2B fibres to atrophy and suggests that female mice are more sensitive to disuse atrophy than male mice. This sensitivity is particularly pronounced in muscles with fast and mixed fibres (Rosa‐Caldwell, Lim, Haynie, Brown, Deaver et al., 2021). When studied, similar sex‐based differences in disuse atrophy are not immediately apparent in humans (Yasuda et al., 2005).

## 4 STRATEGIES TO PROMOTE MUSCLE MASS MAINTENANCE AND MITIGATE DISUSE‐INDUCED MUSCLE LOSS

### 4.1 Pre‐disuse

Although considerable heterogeneity exists among studies investigating the effect of preoperative exercise due to differences in diseases, patients, exercise protocols and assessments, recent evidence suggests that preoperative exercise positively influences postoperative outcomes—reduced length of stay or postoperative complications (Allen et al., 2022; Deprato et al., 2022; Koh et al., 2022). As another model, Master athletes (>60 years) who performed exercise training throughout their life (>20 years) showed prevention of age‐related decline in physical function, including strength (McKendry et al., 2018) and larger type I fibre cross‐sectional area and greater capillary content compared to untrained young individuals (McKendry et al., 2020). In addition, previous investigations in master athletes have shown that exercise training throughout the lifespan exerts a protective effect against declines in some neuromuscular properties (Power et al., 2010)—though that is not always the case (Piasecki et al., 2016). Thus, exercise consistently performed long before the onset of events limiting mobility could attenuate the adverse outcomes caused by disuse, including loss of muscle mass.

**FIGURE 2:**
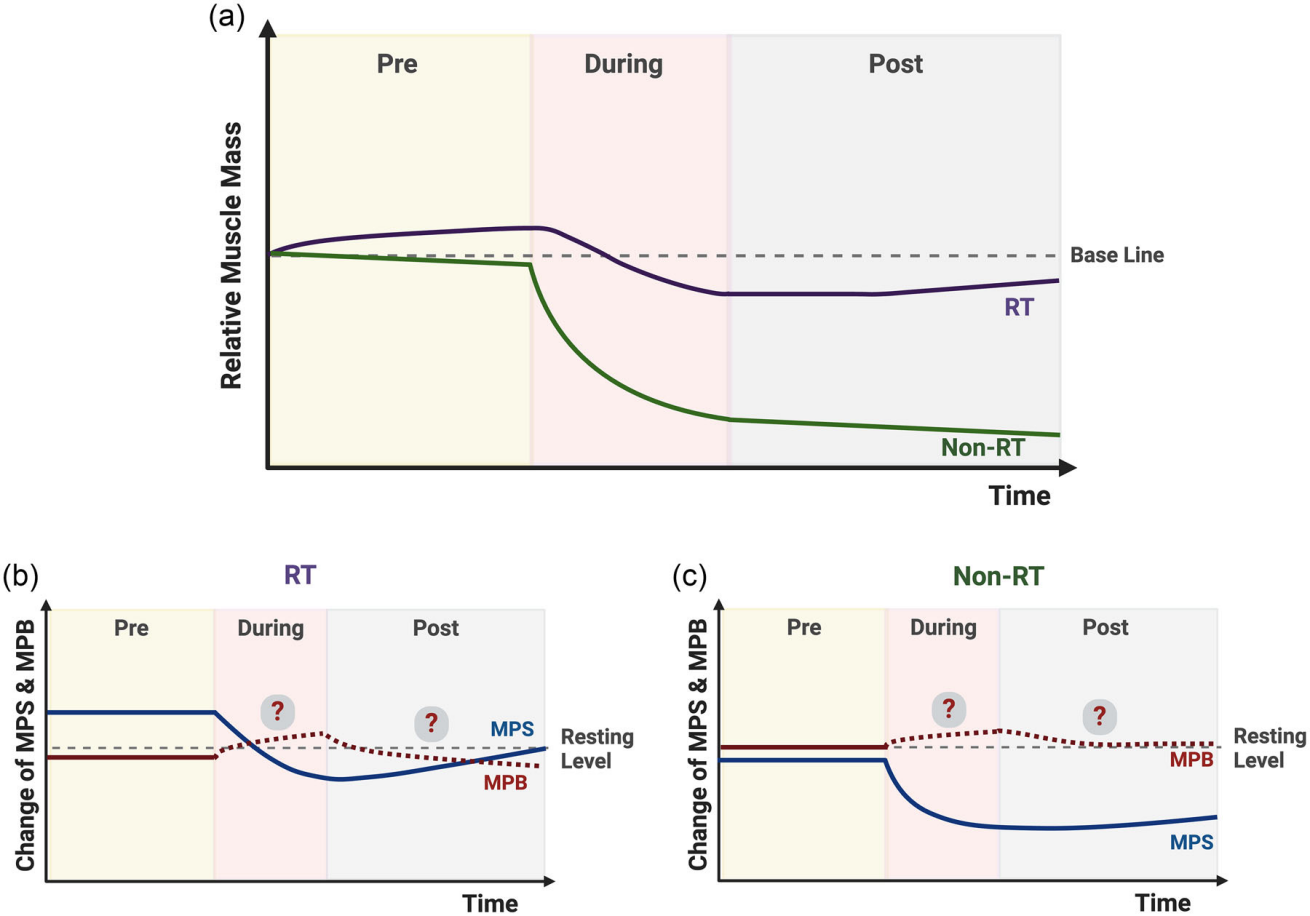
Schematic representation of the changes in muscle mass (a) and protein turnover (b, c) with and without resistance training before, during and following resistance training (McGlory, von Allmen et al., 2018; Smeuninx et al., 2021; Suetta et al., 2009a). Created with BioRender.com.

No study has been conducted to identify the effect of lifelong exercise on disuse atrophy. However, considering the adaptations of longer‐term exercise training—improved microvascular circulation and anti‐inflammatory outcomes, increased muscle mass and enhanced muscle memory (Sharples & Turner, 2023; Snijders et al., 2020)—exercise may be the most effective approach to counteracting sarcopenia and prehabilitating muscle to resist disuse atrophy (Figure 2). However, according to Statistics Canada, only 18% of Canadian adults achieve the recommended amount of physical activity (150 min of moderate‐to‐vigorous physical activity/week). Whilst it may be more (∼60%) in the UK, few individuals still engage in strengthening activities. Thus, regular exercise throughout the lifespan to maintain muscle mass and health should be a primary focus, exercise may offer a short and practical solution to prepare for muscle disuse, such as planned surgeries (Punnoose et al., 2023).

Based on the evidence, aerobic exercise‐type prehabilitation, particularly high‐intensity interval training (Clifford et al., 2023), considering time efficiency, could be a feasible approach to prevent loss of muscle mass by enhancing the supply of nutrients and oxygen during the period of disuse. Enhanced microvascular circulation (e.g., increased capillary content) in skeletal muscle in response to aerobic pre‐conditioning led to greater muscle growth in response to additional RT (Thomas et al., 2022) and activated peripheral endothelial progenitor cells (i.e., angiogenesis), which is one potential mechanism underlying the benefits of preoperative exercise on postoperative outcomes (Esser et al., 2022). On the other hand, RT is the most potent stimulus to promote MPS and induce skeletal muscle hypertrophy (Lim et al., 2022). Indeed, one session of RT before 5 days of bed rest attenuated the decline in MPS and partially prevented disuse atrophy in older men (Smeuninx et al., 2023), and whilst significant heterogeneity in responsiveness to exercise training interventions exists, recent studies have shown that completing an additional exercise volume may be required to overcome non‐responsiveness in older adults (Lixandrão et al., 2024).

Still, there are many unknowns regarding disuse atrophy and the benefits of prehabilitation, including the type and duration of prehabilitation. One pre‐rehabilitation approach that would stimulate the molecular mechanisms of both aerobic and resistance work involves lifting lower loads and completing higher‐volume RT, which could be a promising strategy to alleviate disuse atrophy. Low(er)‐load RT activates MPS, stimulates hypertrophy similarly to higher‐load RT (Currier et al., 2023) and brings about adaptations typically obtained in aerobic exercise (Lim et al., 2019). Furthermore, as RT enhances the sensitivity of muscle to protein ingestion, it would likely be beneficial to pair the RT with a dietary intervention, including a higher‐quality protein diet or other supplements showing benefit to mitigate disuse atrophy, including omega‐3 (McGlory et al., 2019), to maximise the effect.

### 4.2 During disuse

Being adequately prepared for a period of disuse by amassing sufficient muscle mass and strength to enhance resilience to removing the loading stimulus should be the goal for all adults as they age. However, acute disuse events are unpredictable and may be unavoidable, warranting an adaptive approach, and the type of disuse stimulus often dictates the countermeasure employed.

Periods of reduced physical activity/increased sedentary time (e.g., stay‐at‐home orders during a pandemic), where the individual has no restricting illness or injury, is best countered by RT as it remains the most effective strategy to prevent declines in muscle mass and strength (Bamman et al., 1998; Devries et al., 2015; Oates et al., 2010). However, modifications for equipment may be required. Lower‐load RT offers a viable approach to attenuate the negative impact of step reduction on skeletal muscle in older men (Devries et al., 2015). But, in the absence of extensive specialised exercise equipment, home‐based resistance band training or bodyweight exercises are easily implemented and have also been shown to increase muscle anabolism (Marshall et al., 2023), improve body composition (Krause et al., 2019), muscular strength (de Oliveira et al., 2017) and physical function (Liao et al., 2018; Stojanović et al., 2021) in older individuals. Unfortunately, stand‐alone nutritional interventions during reduced physical activity appear to have little impact on muscle mass maintenance (Oikawa et al., 2018), but they may help during recovery.

In situations where individuals undergo a period of ULLI (e.g., broken arm or leg), it may be unsuitable to load the affected limb, at least initially, and alternative strategies are required. If feasible, individuals should be encouraged to maintain activities of daily living and engage in physical activity with modifications where appropriate—to prevent a localised ULLI immobilisation from becoming a systemic disuse stimulus. For example, if someone has a broken arm, they may still be able to exercise by performing lower‐body focused cardiovascular exercise (e.g., running/cycling), bodyweight exercises emphasising the legs and even unilaterally loaded exercises in the contralateral non‐immobilised limb, which may have modest crossover benefits for the strength of the untrained limb (Green & Gabriel, 2018; Manca et al., 2017). However, in some situations, a modified exercise approach may not be feasible, and emphasising nutritional approaches may be the only alternative. Protein or high‐dose amino acid supplementation during ULLI has demonstrated modest efficacy in protecting against the decline in muscle mass (Holloway et al., 2019), though moderate protein doses have not been successful (Backx et al., 2018; Dirks, Wall, Nilwik et al., 2014; Edwards et al., 2020; Mitchell et al., 2018).

Our work shows neither citrulline (Devries et al., 2015) nor Fortetropin (Lim et al., 2023) protects against ULLI‐induced atrophy. Conversely, alternative nutritional approaches have demonstrated promise in protecting against ULLI‐induced muscle losses. High‐dose creatine supplementation (∼20 g/day) protects against upper limb immobilisation‐induced muscle loss (Johnston et al., 2009), possibly due to an impact on MPB (Parise et al., 2001). Also, high‐dose omega‐3 fish oil supplementation attenuated muscle mass declines (McGlory et al., 2019), likely due to attenuating the disuse‐induced decline in MPS (McGlory et al., 2019), modifying the phospholipid membrane composition of skeletal muscle (McGlory et al., 2014) and preventing reductions in mitochondrial respiration (Miotto et al., 2019). Given the vital role that mitochondria play in facilitating skeletal muscle growth with RT (Stokes et al., 2020) and the dysfunction that occurs with ageing (Petersen et al., 2003; Sun et al., 2016) and disuse atrophy (Miotto et al., 2019), countermeasures targeting mitochondrial quality control mechanisms may be an emerging target for tempering atrophy; mitochondrial catalase (Rosa‐Caldwell et al., 2020) and urolithin‐A (Andreux et al., 2019; Luan et al., 2021; Ryu et al., 2016) have shown early promise.

During more systemic disuse events, such as periods of bed rest, including head‐down tilt, which closely mimics hospital admission and space flight, the most feasible approaches are similar to those employed during ULLI. However, neuromuscular electrical stimulation, perhaps in combination with blood flow restriction (Slysz et al., 2021), may also provide an effective countermeasure to disuse‐induced atrophy (Dirks, Wall, Snijders et al., 2014)—though muscle strength is still adversely affected. Nevertheless, implementing effective strategies to offset the musculoskeletal deterioration during the disuse event may go some way to at least ensuring that the recovery period commences from a less compromised position.

### 4.3 Post‐disuse

Following unloading, effective recovery strategies should also be implemented to reclaim the muscle and strength lost during the disuse events. RT is widely accepted as the most effective strategy to counter disuse atrophy. Unfortunately, many older individuals will likely return to their regular physical activity without any attempt to reclaim losses (i.e., passive re‐ambulation), potentially due to the negative consequences of the disuse event, such as pain, reduced mobility and balance issues. However, re‐ambulation following disuse is not always sufficient to restore the loss of muscle mass in younger men (Lim et al., 2023; Mitchell et al., 2018), though in some cases, it may be (Suetta et al., 2009a). Thus, soon after disuse atrophy, RT is almost assuredly necessary for older individuals, and low(er)‐load RT could be a feasible approach to handling impaired physical function.

The biggest challenge is that older adults show very slow or, in some cases, irreversible recovery following periods of disuse despite aggressive RT rehabilitation during recovery (Suetta et al., 2009a). Preclinical studies suggest that the dysregulated metabolic reprogramming and function of aged skeletal muscle pro‐inflammatory macrophages (i.e., reduced succinate, lower HIF‐1α transcription and suppressed glycolysis) (Fix et al., 2021) or impaired protein breakdown, as evident by accumulation of insoluble protein aggregates (Fuqua et al., 2023), may attenuate regrowth during recovery following disuse atrophy. Whilst the mechanisms underpinning this irreversible loss of skeletal muscle in response to disuse atrophy remain uncertain in humans, performing regular RT‐focused rehabilitation for longer periods and with greater volume than previously employed (Lixandrão et al., 2024) may enhance recovery. If RT is combined with appropriate nutritional interventions (e.g., proteins, creatine, omega‐3), recovery from disuse may be accelerated. Additionally, the longer‐term, regular RT will likely lead to other health benefits (e.g., mobility, cognitive function and metabolic health) beyond the restoration of muscle mass (Abou Sawan et al., 2023).

## 5 RESISTANCE TRAINING PRESCRIPTIONS AND THE PHYSIOLOGICAL BENEFITS IN THE ELDERLY

**FIGURE 3:**
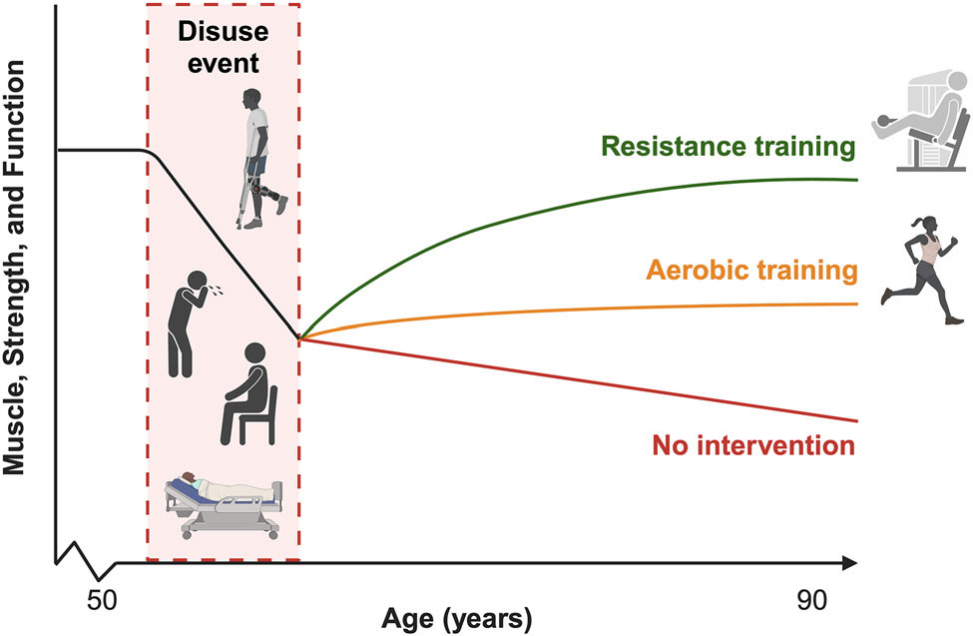
Schematic representation of exercise training modalities following a period of muscle disuse to recover skeletal muscle mass, strength and function (e.g., gait speed) in older individuals (Bamman et al., 1998; Schroeder et al., 2019; Villareal et al., 2017; Willis et al., 2012). Created with BioRender.com.

Historically, aerobic exercise has been the most widely recommended form of exercise as it reduces risks of all‐cause mortality, cardiovascular disease and cancer (Bull et al., 2020). The guidelines also include two weekly bouts of strengthening work; however, older adults often omit RT (Sandercock et al., 2022). RT is the most effective treatment to mitigate disuse‐induced skeletal muscle atrophy as it improves muscle hypertrophy, strength and power (Figure 3) (Suetta et al., 2004). But, RT has several other physiological benefits, including cardiorespiratory, vascular and mental health (Shailendra et al., 2022), all of which contribute to mitigating frailty and improving mobility and independence for older adults (Fiatarone et al., 1994). Recent evidence suggests RT can be as effective as aerobic exercise in reducing cancer risks, delaying mortality, mitigating the progression of chronic diseases and preventing functional declines (Chen et al., 2021; Mende et al., 2022). However, all‐cause mortality is lowest when resistance and aerobic exercise are performed concomitantly (Brellenthin et al., 2022).

Whilst RT may attenuate the negative effects of age‐related diseases, including sarcopenia and frailty (Talar et al., 2021), prescriptions for RT may depend on the individual's personal goals. Almost any load, for many repetitions, multiple times a week may be better for building muscle mass (i.e., hypertrophy), whereas higher loads and fewer repetitions enhance muscular strength to a greater degree (Currier et al., 2023). Minimal dose approaches, such as higher‐load at low frequencies or lower‐load at higher frequencies, may be equally effective in delaying the onset of sarcopenia and age‐related atrophy (Fyfe et al., 2022). For example, two weekly exercise sessions focused on upper‐ and lower‐body exercises performed with a relatively high degree of effort for 1–3 sets of 6–12 repetitions (Hurst et al., 2022). Importantly, implementing progressions (i.e., progressive overload) is critical to ensure continued strength and hypertrophy improvements over time, and the rating of perceived exertion (RPE) scale may be the preferred approach for older adults to include during RT (Buskard et al., 2019).

Older adults should consider using lower‐load RT to improve clinically important outcomes. Recent studies have demonstrated that lower‐load RT induces similar hypertrophy and strength gains as higher‐load RT (Carvalho et al., 2022; Schoenfeld et al., 2017). Low(er)‐load RT may benefit older adults by maximising benefits and safety whilst minimising barriers to participation. In line with this, employing resistance bands may be a desirable approach for low(er)‐load RT, particularly as resistance band training has been shown to activate quadriceps muscle similarly to regular (machine‐based) RT (Marshall et al., 2020). However, long‐term training studies in older individuals using resistance bands are needed to confirm this. Together, we suggest an exercise approach to mitigate age‐related muscle disuse atrophy would include participating in RT at least twice weekly, performing multiset exercises, using lower‐to‐higher loads, with a high degree of effort and emphasising progression through increased volume of work or loads with advances in strength. Pairing this with regular activity and reduced sedentary time will help older adults offset the trajectory of abrupt disuse‐induced declines in muscle mass and strength, maintain their functional independence and promote their health span.

## 6 CHALLENGES TO IMPLEMENTATION

Despite projections that a modest ∼10% decrease in lower muscle (handgrip) strength prevalence would save ∼$546 million per year (Chaput et al., 2023), RT is not currently prescribed to older adults experiencing disuse. Physiotherapists report several perceived barriers to prescribing RT to older adults in acute care, including a lack of prioritisation and a clear definition of RT, insufficient support personnel and perceived poor patient motivation (Chan et al., 2022). Patients’ capacity, therapists' confidence, and equipment are commonly reported barriers to prescribing RT for older adults (Williams & Denehy, 2019). Amongst community‐dwelling older adults and individuals with chronic conditions, multi‐component, progressive group‐based training programs are cost‐effective (Barbosa et al., 2022; Guillon et al., 2018; Subias‐Perie et al., 2022) and can be effectively implemented (McKay et al., 2018; Petrescu‐Prahova et al., 2016). Support from the government, trained staff, accessibility of programs and financial resources are critical factors in the successful implementation of physical activity programs for older adults (Sims‐Gould et al., 2019).

Older adults describe experiencing personal barriers when participating in physical activity. Environmental factors and resources are commonly identified barriers (Spiteri et al., 2019). Similarly, older adults in the UK report not being aware of the strength components of the current physical activity guidelines compared with the aerobic components (Gluchowski et al., 2022). Older adults believe walking, yoga and Pilates qualify as progressive strength exercises (Gluchowski et al., 2022). Others have documented that older persons believe physical activity is unnecessary or potentially harmful (Franco et al., 2015). Regardless, RT programs must be designed and implemented around social and connectedness motivators to engage older adults and improve their adherence. Therefore, knowledge mobilisation and implementation are urgently required to aid older adults participating in RT to mitigate age‐related disuse atrophy.

In summary, the loss of muscle in response to periods of unloading is a highly coveted topic in physiological research that will, and should, continue to harness attention from researchers worldwide for many years. Several advancements have been made in understanding the mechanisms driving disuse‐induced atrophy; however, individual protein and post‐translational modification analysis—particularly by way of the ‘omics revolution—will enhance the resolution with which we can continue to unpick muscle atrophy's intricacies. As it stands, RT—before, during and after—is our best form of defence against the perils of unloading, but designing and implementing effective RT programs in older individuals comes laden with challenges.

## AUTHOR CONTRIBUTIONS

All authors have read and approved the final version of this manuscript and agree to be accountable for all aspects of the work in ensuring that questions related to the accuracy or integrity of any part of the work are appropriately investigated and resolved. All persons designated as authors qualify for authorship, and all those who qualify for authorship are listed.

## CONFLICT OF INTEREST

J.M., G.C., E.A.N and C.L. declare no conflicts of interest. S.M.P. reports grants or research contracts from the US National Dairy Council, Canadian Institutes for Health Research, Cargill, Friesland Campina, Dairy Farmers of Canada, Roquette Freres, Ontario Centre of Innovation, Nestle Health Sciences, Myos, National Science and Engineering Research Council, and the US NIH during the conduct of the study; personal fees from Nestle Health Sciences, non‐financial support from Enhanced Recovery, outside the submitted work. S.M.P. has patents licensed to Exerkine but reports no financial gains from patents or related work.
